# Substrate Induced Strain Field in FeRh Epilayers Grown on Single Crystal MgO (001) Substrates

**DOI:** 10.1038/srep44397

**Published:** 2017-04-12

**Authors:** C. W. Barton, T. A. Ostler, D. Huskisson, C. J. Kinane, S. J. Haigh, G. Hrkac, T. Thomson

**Affiliations:** 1 NEST Research Group, School of Computer Science, The University of Manchester, Oxford Road, Manchester, M13 9PL, UK; 2 Université de Liège, Physique des Matériaux et Nanostructures, Liège, B-4000 Sart Tilman, Belgium; 3 College of Engineering, Mathematics and Physical Sciences, Harrison Building, Streatham Campus, University of Exeter, North Park Road, Exeter, EX4 4QF, UK; 4 School of Materials, The University of Manchester, Oxford Rd, Manchester M13 9PL, UK; 5 ISIS, Harwell Science and Innovation Campus, Science and Technology Facilities Council, Rutherford Appleton Laboratory, Didcot, Oxon OX11 0QX, UK

## Abstract

Equi-atomic FeRh is highly unusual in that it undergoes a first order meta-magnetic phase transition from an antiferromagnet to a ferromagnet above room temperature (*T*_*r*_ ≈ 370 K). This behavior opens new possibilities for creating multifunctional magnetic and spintronic devices which can utilise both thermal and applied field energy to change state and functionalise composites. A key requirement in realising multifunctional devices is the need to understand and control the properties of FeRh in the extreme thin film limit (*t*_*FeRh*_ < 10 nm) where interfaces are crucial. Here we determine the properties of FeRh films in the thickness range 2.5–10 nm grown directly on MgO substrates. Our magnetometry and structural measurements show that a perpendicular strain field exists in these thin films which results in an increase in the phase transition temperature as thickness is reduced. Modelling using a spin dynamics approach supports the experimental observations demonstrating the critical role of the atomic layers close to the MgO interface.

FeRh undergoes a first order metamagnetic phase transition from an antiferromagnetic (AFM) phase to ferromagnetic phase (FM) when such stimuli such as thermal or strain energy are introduced to the system. Originally discovered by Fallot (1937) in studies of Fe based intermetallic alloys of Ru^1^, Ir^2^ and Rh^3^, later work by Kouval (1962) further demonstrated that a magnetic phase transition could be observed when the alloy was heated above room temperature^3^,^4^,^5^,^6^.

Fe_x_Rh_1−x_, when grown close to equi-atomic (48 ≤ x ≤ 56 at% Fe) composition, forms a B2 CsCl-type chemically ordered binary alloy. When correctly grown this CsCl type structure will exhibit an antiferromagnetic phase, denoted *α*″-FeRh, at room temperature. It has been shown that this AFM phase is a type-II or G-type antiferromagnet, such that adjacent lattice planes parallel to the {111} CsCl planes are antiferromagnetically coupled with respect to each other and ferromagnetically coupled within the plane^7^ as shown schematically in Fig. 1(a) inset. The phase transition can be induced by subjecting the FeRh system to external stimuli including temperature^3^, pressure^8^, applied magnetic^9^,^10^,^11^ and electric fields^12^, femtosecond laser pulses^13^,^14^,^15^ and more recently spin polarized currents^16^.

The physical properties of FeRh have recently lead to an intensified interest in this system due to its potential to be incorporated into functional devices. For example, manipulation and control of magnetic order in FeRh via application of an electrical field^17^ and spin polarized current^18^ has demonstrated promise in low power spin based electronics for storage and artificial multiferroic based logic applications^19^,^20^. Very recent studies have also demonstrated that the first order meta-magnetic phase transitions in FeRh alloys can be induced by manipulating the atomic separation using ferroelectric substrates such as (001) oriented ferroelectric PMN-PT^17^ or BaTaO_3_^12^,^21^.

In order for FeRh to be included in real world devices, traditional scaling arguments^22^,^23^ imply that very thin layers will need to be employed. The majority of previous work published on FeRh has focused on bulk-like properties in films on the order of several 10’s of nm and above^7^. However, more recent work has investigated effects in low dimensional epilayers of FeRh^24^,^25^,^26^,^27^ where it is known that interface regions exhibit symmetry breaking and low dimensionality that can significantly modify the physics of the system^28^. However, an understanding of the role of interface effects on the properties of the phase transition and how this may limit future FeRh based devices is still lacking.

In this work we demonstrate the methodology needed to create sub-10 nm thin films of FeRh on single crystal MgO substrates, which then allows the properties of FeRh in the thin film limit to be explored systematically. Experimental results are compared with simulations to develop a deeper understanding of the MgO/FeRh interface and demonstrate how the AFM/FM transition changes with varying film thickness which potentially sets a fundamental limit on FeRh layer thickness.

## Results: Effect of film thickness

**Figure 1:**
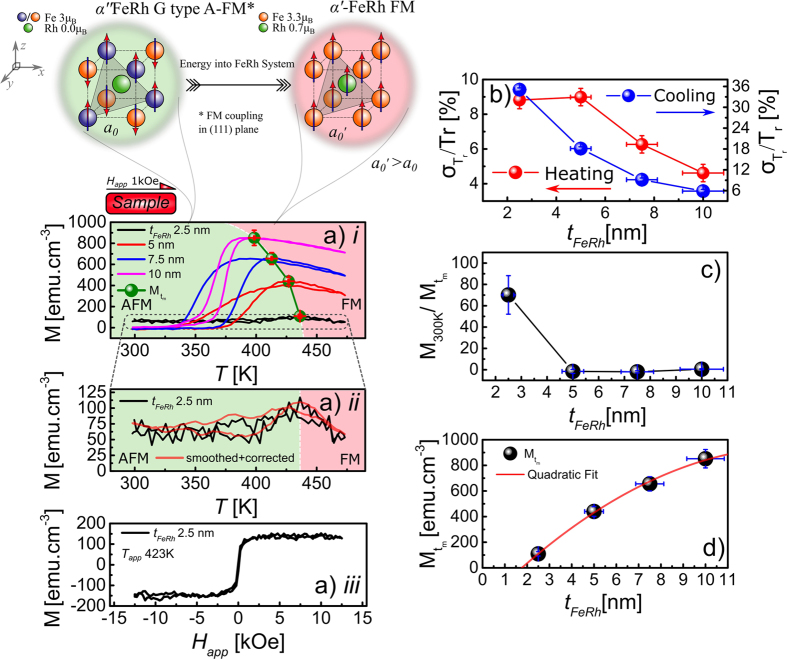
Magnetometry obtained by VSM showing the effect of film thickness of t_FeRh_; (**a**) i thermal hysteresis loops performed with an inplane field of 1kOe showing the AFM(green) to FM(red) regions. Also plotted is the maximum point of the phase transition 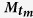 as a function of the temperature at which that this occurs ***T***_***m***_; (**a**) ii shows the thermal hysteresis for the 2.5 nm film along with the 10pt Savitzky–Golay filtered and drift corrected data; (**a**) iii shows the M-H data for the 2.5 nm film taken at an average a temperature of 423 K. The outset (above) shows schematically the magnetic order of the Fe sub-lattices before and after the phase transition in addition to the induced moment on the Rh atom; (**b**) the extracted normalised transition temperature distribution as a function of film thickness during the heating and cooling cycles; (**c**) ratio of the magnetization before, ***M***_**300*****K***_, and after at the maximum point of the phase transition 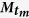 and (**d**) 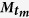 as a function of film thickness fitted with a quadratic function.

In this work a set of MgO/Fe_50_Rh_50_ samples, where the thickness (*t*_*FeRh*_) ranged from 2.5 nm to 10 nm were produced. Since the purpose of our work was to investigate the effect of the substrate/FeRh interface, no capping layer was used and hence the samples contained a single MgO/FeRh interface capable of exerting a crystallographic strain on the magnetic thin film. Magnetometry was used to investigate the meta-magnetic phase transition as a function of temperature. Figure 1(a) i and 1(a) ii show the thermal hysteresis loops, from which it is evident that as the film thickness is reduced the phase transition undergoes a significant change where the nucleation and transition temperatures shift to higher temperatures. The ferromagnetic nature of the 2.5 nm film is confirmed by Fig. 1(a) iii which shows the hysteresis (M-H) loop measurement at T = 423K. The change in the transition temperature and width is summarized in Fig. 1(b), which shows the normalised phase transition width 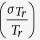
where 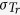 is the standard deviation of the fitted transition width (obtained from dM/dT) and *T*_*r*_ is the temperature taken at the mid-point of the transition. Figure 1(a) i shows that the transition moves to higher values as the thickness is reduced for both heating and cooling cycles. Figure 1(b) demonstrates the asymmetry between the heating (AFM/FM) and cooling (FM/AFM) cycles which is similar to that typically observed for FeRh films approximately ≤50 nm^29^. It is also apparent from the thinnest (2.5 nm) sample that the transition to the AFM phase is incomplete as indicated by the presence of the FM signal below the transition. Stabilized ferromagnetism at FeRh/MgO interfaces can be understood as arising from anisotropic^30^ graded^31^ strain fields along the substrate film normal, which act to change the magnetic order of the strained region in addition to other interface related phenomena^30^. This is demonstrated in Fig. 1(c), where the ratio of the magnetization prior to and directly following the phase transition is plotted as a function of film thickness. This ratio is essentially zero for the films with thickness ranging from 10 nm–5 nm. However, in the thinnest sample the ratio dramatically increases to ≈70% due to the relatively large magnetization at 300 K and the reduced magnetization at full saturation. These data agree qualitatively with similar data in the literature^25^. Figure 1(d) shows the maximum value of magnetisation during the M vs. T curves (phase transition) 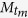 as a function of film thickness. These data strongly suggest the presence of a region of material that does not contribute to the measured magnetic signal, as the intercept in *t*_*FeRh*_ is non-zero. Fitting the experimental data with a 2^nd^ order polynomial gives a value for the thickness of this region of 1.76 ± 0.15 nm.

**Figure 2:**
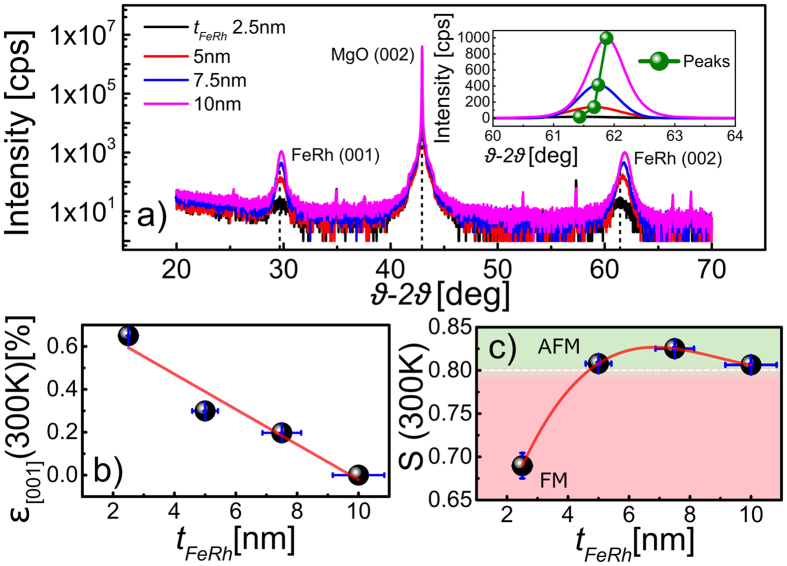
Structural analysis obtained by XRD in Bragg-Brentano geometry showing; (**a**) θ–2θ spectra for the thickness ranges 10 nm–2.5 nm, inset peak fits shows the shift in the FeRh (002) diffraction peak as the thickness of the film is reduced (highlighted are the (001) and (002) FeRh peaks); (**b**) shows the calculated perpendicular strain at 300K as a function of the film thickness relative to the 10 nm FeRh film (red line is a guide to the eye) and (**c**) shows the extracted ordering parameter S calculated from the integrated intensity of the fundamental (001) and superlattice (002) peaks, the shaded regions represent the films that exhibit AFM (green) or FM (red) behavior at 300K (red line is a guide to the eye).

As the magnetic properties of FeRh thin films are intrinsically linked to the crystal structure, x-ray diffraction (XRD) measurements were undertaken to investigate their crystallographic and texture properties. The XRD measurements were performed in the standard *ϑ*–*2ϑ* geometry where the observed diffraction is due to crystallographic planes parallel to the substrate surface, i.e. perpendicular scattering vector (q_z_). Figure 2(a) shows the measured *ϑ*–*2ϑ* diffraction spectra for the thickness series. These data demonstrate a well formed B2 CsCl type structure shown by the presence of the FeRh (001) fundamental and (002) superlattice peaks. The data also demonstrate that as the thickness of the samples is reduced the relative intensities and the positions of the peaks change. This is highlighted in the inset where it can be seen that the peak position moves to lower angles as the film thickness is reduced (green line in insert). The perpendicular lattice constant *c* (along [001] denoted *c*_*001*_) was extracted from the position of the (002) superlattice peak and compared to the value of the lattice parameter of ≈2.997 Å for the 10 nm thick film denoted as *c*_*0*_. This allows the strain along the, [001] direction normal to the film, *c*_*001*_, to be quantified as a function of film thickness. The strain *ε*_*001*_ was calculated using equation (1),


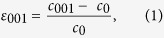


From Fig. 2(b) it is clear that as the film thickness is reduced, there is a corresponding increase in the perpendicular strain. This increase is monotonic between 10 nm–2.5 nm, with a significant increase in the strain of ≈0.65% for the 2.5 nm relative to the 10 nm film.

The XRD measurements of the (001) and (002) peaks also allow the degree of chemical order to be estimated. The chemical order parameter *S*, is a measure of the fraction of Fe/Rh lattice sites that obey the ordering conditions of the Fe/Rh atoms^32^. It can be calculated^33^ using 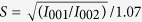 where 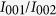 are the integrated intensities of the fundamental and superlattice peaks respectively. Figure 2(c) plots *S* as a function of the film thickness. These data demonstrate that as the thickness is reduced, the structural ordering *S*, within error, remains constant at ≈0.81 for films in the range the 10–5 nm. However, as the film thickness is reduced to 2.5 nm *S* decreases to ≈0.68.

**Figure 3:**
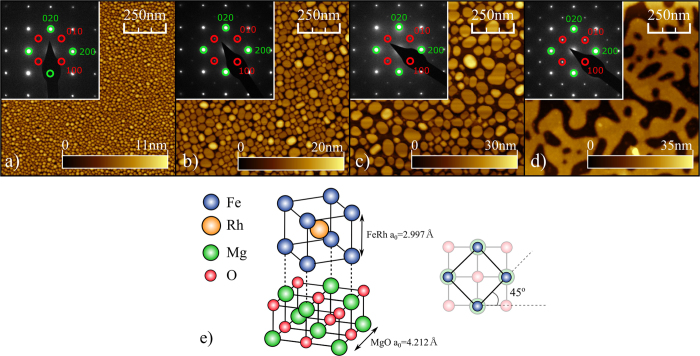
Atomic force micrographs and selected area electron diffraction patterns showing; (**a–d**) 1 μm scans of the FeRh films as a function of increasing thickness which demonstrates the formation of island growth due to strain relaxation and formation of dislocation leading to Volmer-Webber type growth. The inserts show the indexed TEM diffraction patterns in which green is used to highlight the 020/200 spots from the MgO phase and red to highlight 010/100 spots from the FeRh. (**e**) Schematic representation of the relative orientations of the two lattices with the FeRh B2 lattice orientated along the [110] inplane direction, 45° misoriented relative to the MgO cubic [200] direction.

The surface structure of these FeRh thin films was also investigated using atomic force microscopy and transmission electron microscopy (TEM). Figure 3(a–d) shows atomic force microscopy data that reveals a Volmer-Webber island type growth mode is present in these films as previously observed in FeRh films in a similar thickness regime^24^,^26^. Furthermore, it is found that the island size varies as a function of the film thickness. Figure 3(a–d) demonstrates that initially a nano-island topography exists where the island size is ≈20 nm for the 2.5 nm film which then grows laterally until coalescence between the islands, resulting in a stripe type pattern for the 10 nm film. This evolution of the film topography can be understood in terms of the relative binding energies between that of the film and the MgO substrate^24^,^26^. The insets of Fig. 3 show TEM selected area diffraction (SAED) patterns which reveal a clear cubic epitaxial relationship between the FeRh film and the MgO substrate, where the unit cell of the FeRh is rotated 45° in-plane relative to the unit cell of the MgO, such that MgO [200] is parallel to FeRh [110], shown schematically in Fig. 3(e). As the thickness of the film decreases, the intensity of the FeRh 100 and 010 SAED spots decreases, disappearing entirely for the thinnest sample. We hypothesise that this is not simply due to the decreasing volume, but is additionally attributed to the decreasing chemical ordering of the FeRh film when close to the MgO surface and an increased presence of perpendicular lattice strain in the film.

## Atomistic Simulations/Modelling

In an attempt to understand the underlying mechanism for the observed increase in the phase transition temperature with decreasing film thickness, we have performed atomistic spin dynamics simulations of FeRh films of various thicknesses. We have included within the model of the FeRh films a 1.5 nm interface layer to approximate the experimental results. By performing a systematic variation of the possible magnetic orderings within this layer we can obtain an insight into the nature of its magnetic structure.

We use the atomistic spin dynamics model^34^ that treats each atomic magnetic moment as a localized atomistic spin and is based on a Heisenberg Hamiltonian formalism that, in this case can be written as below:


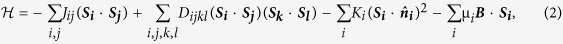


Here *J*_*ij*_ and *D*_*ijkl*_ are the bilinear and four spin terms that take the values given in *Barker et al*.^35^. We take the anisotropy constant, *K*_*i*_, from *Mancini et al*^36^. These authors measured four anisotropy constants but here we include only the largest term which was shown to be more than two orders of magnitude greater than the others. The largest energy contributions in the Hamiltonian, eq. (2), are the bilinear and four-spin terms. The bilinear terms have ferromagnetic nearest and second nearest neighbor terms. The bilinear terms support the ferromagnetic state, whereas the next nearest neighbor terms supports antiferromagnetic order. The four spin term gives rise to antiferromagnetic order and has a different temperature scaling to the bilinear term. This allows the phase transition to arise due to the competition between the exchange interactions.

**Figure 4:**
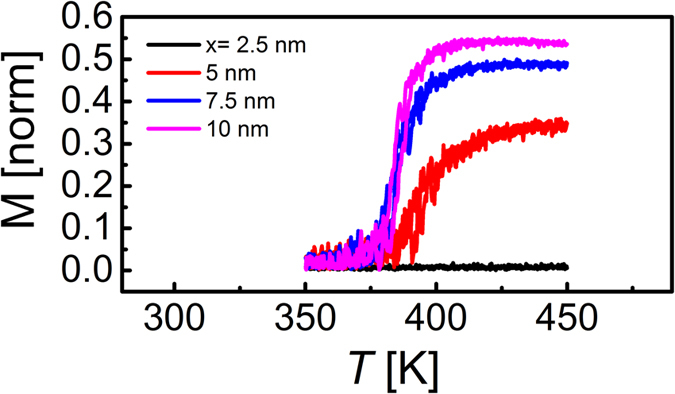
Numerically determined thermal hysteresis loops as a function of FeRh thickness (data offset for clarity), with a 1.5 nm antiferromagnetic layer and the total thickness shown by the labels. As the thickness of the layers is decreased there is an increase in the transition temperature consistent with the experimental measurements of Fig. 1(a). For the 2.5 nm case there is no detectable phase transition.

The temperature dependent, quasi-equilibrium, magnetization is found by solving the many body Landau-Lifshitz-Gilbert equation of motion^37^. In order to create a feasible regime for the numerical simulations, used to determine the thermal hysteresis loops, a heating/cooling rate of 50 K/ns was employed. This rate is much faster than used experimentally in magnetometry measurements. To compensate for this, we solve the Landau-Lifshitz-Gilbert equation in the critical damping regime to drive the system more rapidly to the ground state. We have simulated the thickness dependence of FeRh with an interfacial AFM layer, with an FM layer and without any interfacial layer. We determine that for the latter two simulations, (with an FM layer and with no layer) the phase transition temperature always decreases as the sample thickness is reduced. This is contrary to our experimental findings; therefore we conclude that neither of these hypothetical situations represents the magnetic state of our samples. Only the simulation with the antiferromagnetic exchange layer, the 1.76 nm region that does not contribute to the magnetic signal, as shown in Fig. 1(d) is able to reproduce the increase in the phase transition temperature with sample thickness seen in Fig. 1(ai). The numerically determined thermal hysteresis loops^38^ calculated for an AFM layer, Fig. 4, show good qualitative agreement with our experimental data in Fig. 1. Our XRD results demonstrate an increasing crystal strain in the thinnest films, believed to be associated with the FeRh/MgO interface. Such strain can significantly alter the magnetic ordering^39^ and we propose that this is the reason for antiferromagnetic exchange in the layer near the FeRh/MgO interface.

The presence of antiferromagnetic order in the AFM interface layer favors the formation of the antiferromagnetic phase in the remaining part of the FeRh film. The exchange in this layer must be sufficiently strong to overcome the partial frustration at the interface, as the FeRh moves towards the ferromagnetic state. Otherwise, the transition temperature will decrease with decreasing thickness.

## Strain based two-layer model

**Figure 5:**
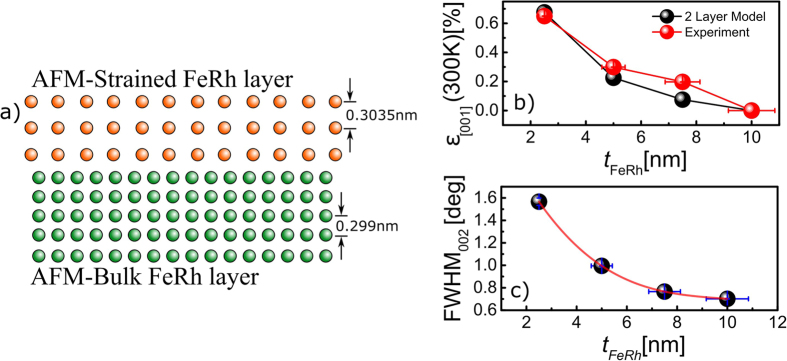
Overview of the proposed two-layer strain model showing (**a**) schematic representation of the AFM layer (red atoms) and B2 CsCl FeRh (green atoms); (**b**) compares our theoretical model for a 1.5 nm strained AFM layer with the experimental strain data obtained from XRD; and (**c**) extracted width data from the (002) FeRh peak showing the FWHM value as a function of thickness (red curve is a guide to the eye).

Atomistic spin dynamics modelling provides a detailed atomic level simulation of the ultra-thin FeRh films and our results show that the increase in the transition temperature can be explained by assuming that there is a thin, 1.5 nm, AFM coupled layer adjacent to the MgO substrate. This AFM coupling acts to support a strained *α*″ AFM B2 structure requiring higher temperatures to overcome the phase transition as the film thickness is reduced. Whilst this modelling provides a comprehensive description of the system, it is also extremely useful in developing technological applications to provide a simpler description which allows the essential features of the magnetic properties to be understood in a more intuitive manner. Here we present a simple two-layer model based on strain differences. In this model the two layers represent; *i)* the layer observed in the magnetometry data, approximately 1.76 nm, that did not contribute to the magnetic signal and *ii*) the bulk *α*″ AFM B2 phase which represents *t*_*FeRh*_ − 1.76 nm of the film, Fig. 5(a). The lattice constants of the two layers were chosen such that the total weighted average approximately equaled the measured lattice constant for that particular layer, therefore, these constants were set as 0.3035 nm and 0.299 nm for the *α*″ AFM strained layer and *α*″ AFM bulk like layer respectively. The calculated strain was determined according to eq. (1) using the weighted average calculated lattice constant and normalized to the calculated lattice constant at 10 nm. In this two layer model we again assumed the AFM strained FeRh layer to be 1.5 nm to approximate the layer measured magnetically. Figure 5(b) shows the results from the two-layer model for a 1.5 nm strained layer along with the measured strain as a function of thickness for comparison. This simple model agrees well with the experimental data providing a useful insight into the physical origin of the magnetic behavior. In reality, we expect the situation to be more complex than can be described by just two layers. This is suggested by the increase in the width of the transition as the film thickness is reduced indicating the presence of additional strain as shown in Fig. 5(c), where additional peak broadening is observed. In the model, the AFM layer is interpreted as a region with an increased lattice constant where the AFM coupling is strongest. Hence, as the film thickness is reduced, the film is comprised of a portion of the AFM layer and the strained region leading to a larger measured lattice parameter.

## Discussion

In this work we have demonstrated that the magnetic properties of FeRh thin films are highly dependent on film thickness. Magnetometry revealed that as the film thickness is reduced from 10 nm to 2.5 nm the first order metamagnetic phase transition undergoes a pronounced systematic variation. We find that there is a large increase of ≈40 K in the transition temperature and a broadening of the thermal hysteresis. We attribute the changes in the magnetic behavior, importantly the increase in *T*_*r*_, to the structural changes measured by XRD, where it is shown that as the film thickness is reduced there is an increase in the perpendicular strain, along the [001] direction, such that the change in the latent heat energy required to nucleate the phase transition increases. This conclusion is further supported by recent *ab initio* electronic simulations which show that the body centred tetragonal (bct) expanded tetragonally strained structure is likely to be the ground state configuration for FeRh 40. This could explain why we observe an increase in the transition temperature as the FeRh is increasingly strained as the thickness is reduced. We attribute the observed lattice expansion to the presence of a large interface strain field that acts along the [001] direction which is more significant in the thinnest films of FeRh due to its limited perpendicular extent. The net result of this strain is a film containing a hybrid of two phases of FeRh; the *α*″ AFM B2 structure as indicated by the presence of the fundamental (001) peak and a strained FeRh later which exhibits a larger AFM coupling, due to the larger lattice parameter, than is exhibited by the majority of the FeRh film. In the thinnest film a small (compared with fully ordered FeRh) ferromagnetic moment persists below the expected transition. XRD data reveals an increase in the strain as the film thickness is reduced which is accompanied by a significant reduction in the chemical order parameter *S*. The ground state energies of FeRh are known to be highly sensitive^30^ to the termination layer element^41^, especially in thin films of the order of 5–8 monolayers. No evidence of in-plane strain accommodation, within the detectable limit of ±0.5% was found through analysis of TEM selected area diffraction patterns for the films spanning the range 10 nm–5 nm. The lack of detectable in-plane strain accommodation indicates that the FeRh lattice is locked into position along the [110] direction at the MgO/FeRh interface and that the film is allowed to expand only along direction [001], consistent with the XRD data. The Volmer-Webber type growth, observed by atomic force microscopy offers an ideal platform to study the fundamental limits and physics of FeRh thin films grown in the ultrathin limit. This is demonstrated through the epitaxial growth of the B2 FeRh onto (001) MgO substrates forming islands of highly-ordered B2 CsCl FeRh which is highly textured.

## Conclusions

In conclusion, we demonstrate experimentally that reduced dimensional epilayers of FeRh contain a graded perpendicular strain fields at the MgO/FeRh interface. We show that this strain affects the nucleation of the first order meta-magnetic phase transition. As the thickness of the films is reduced, the strain has a greater effect, increasing the energy barrier for phase nucleation and resulting in the start of the phase transition being shifted towards higher temperatures. Accompanying this increase, we observe an increase in the transition width and reduction in the ordering parameter *S,* which could set a fundamental limit on the thickness of these ordered binary alloys. Using atomistic modelling we demonstrate that the effect of the strain is to induce an antiferromagnetic layer, which supports the antiferromagnetic state in the FeRh layer resulting in an increased transition temperature with decreasing thickness. These results provide good evidence that the transition temperature and width of the thermal hysteresis can be controlled using appropriate choices of seedlayers where both the lattice constant and thermal expansion coefficient must be carefully matched to FeRh. We also introduce a simpler two-layer model that allows essential features of the interfacial layers to be described in a manner that can be easily understood and incorporated into models of larger systems, for example in simulations of heat assisted magnetic recording or spintronic devices.

## Methods

### Sample Growth and Preparation

The samples were grown by dc magnetron sputtering using an 11 target AJA sputter system from a Fe_50_Rh_50_ alloy target, with a base pressure of better than 5 × 10^−9^ Torr, a process pressure of Ar of 3 mTorr and a gun power of 100 W. All samples were deposited on 10 × 10 mm single crystal (001) oriented MgO substrates. A substrate temperature T_Sub_ of 650 °C was used during film deposition, this temperature was then increased to 750 °C for post-deposition annealing. The films were left to cool under vacuum, until ambient conditions were established. Two samples were grown for each set of conditions, one of which was used for the Atomic force microscopy study and X-ray diffraction measurements. The other was then cut into a nominally 8mm disk using a South Bay Technology Model 360 disk cutter. The remaining material was then used to fabricate the Transmission electron microscopy wedge samples.

### Magnetic Measurements

The magnetic properties of the FeRh thin films were measured using a MicroSense model 10 vector vibrating sample magnetometer (VSM). The temperature hysteresis measurements were performed in a temperature range of 298 K–473 K with a step size of 3 K using a soak period of 60 s and a sampling average 50. This resulted in an effective temperature sweep rate of 1.17 Kelvin/minute. A 1 kOe inplane applied magnetic field was used to saturate the domain structure along the film plane during the measurements and signals were measured both parallel and perpendicular to this plane. Background subtraction was performed by measuring, under the same conditions, a bare MgO substrate which was then subtracted from the measurement of the film. A 10 point Savitzky–Golay digital filter was used to help enhance the signal-to-noise ratio (SNR) whilst maintaining the measured signal. From the smoothed data all analysis was performed. Volume normalisation of the measured magnetic signal was performed using the nominal thickness and the disk dimensions as measured by a digital Vernier caliper. Error considerations where taken from uncertainties in the film thickness, disk radius temperature measurement and magnetic moment. In order to extract the transition temperature *T*_*r*_, transition width σ_Tr_ and magnetisation at the maximum point of the phase transition 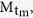 a Boltzman growth function summed with a quadratic function was used to fit the smoothed data from which parameters were extracted either directly from the fits or by differentiation of the processed data which were then fitting with a Gaussian function. The magnetization at 300 K was extracted by using a cubic fit of the data about 300 K. The magnetic hysteresis (M-H) data for the 2.5 nm sample was taken at an temperature of 423 K, using a field step size/sweep rate of 250 Oe and with a sampling average of 70.

### Structural Analysis

Structural analysis was undertaken by X-Ray diffraction (XRD) and transmission electron microscopy (TEM).

The XRD data were collected on a Rigaku Smartlab X-Ray diffractometer equipped with a 9 kW rotating anode and operating at the CuK_α1_ (λ = 1.540593(2)Å) wavelength obtained via a Ge (220) double bounce monochromator. The stage used in this experiment was an Anton-Parr furnace. The step size used in these measurements was 0.01 degrees and the *2ϑ* range was 20–70 degrees at a rate of 0.6 degrees/minute. In order to extract the structural ordering parameter S, lattice constant *c* and peak parameters such as the FWHM value, a 10 point Savitzky–Golay digital filter was used around the (001) and (002) peak positions. Following this, a Pearson VII peak function was fitted to the smoothed data which included a linear background, this allowed values such as peak position, integrated intensity and FWHM to be determined. Lattice parameter determination, hence strain calculations, were carried out using the (002) FeRh peak in order to reduce the effective angular resolution limit which varies as 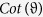 allowing for higher accuracy measurements to be taken, as given by the differential Bragg equation. Errors in the lattice parameter, hence strain, where obtained through propagation of the angular resolution, assumed to be approximately 0.03 degrees, with the spectral bandwidth uncertainties. Uncertainty in the ordering parameter was taken from the errors in the areas of the fitted diffraction peaks.

TEM imaging was performed with an FEI Tecnai T20 TEM using a LaB_6_ source and an accelerating voltage of 200 kV. The samples were thinned to electron transparency by mechanical polishing of the substrate using an Allied High Tech Multiprep automatic polishing machine. This uses a variation on the tripod polishing technique which is required for preserving the crystallinity of the samples, avoiding the surface amorphisation layer associated with ionic polishing techniques. The lattice parameter of FeRh was determined from the SAED patterns using the MgO substrate as a built-in standard. The measurement was optimized by employing a broad parallel electron beam over a large sample area to ensure small sharp diffraction spots and by taking repeat measurements from each pattern along both <100> and <110> type directions, considering both low order and higher order spots. In this way the accuracy of strain measurement was estimated to be better than 1%. The SAED patterns displayed in Fig. 3 have been cropped from the original data for clarity, and the MgO and FeRh spots are highlighted in green and red respectively.

### Surface Analysis

Atomic force microscopy was used to investigate the surface topography. These measurements were taken on a Bruker Dimension Icon instrument in peakforce tapping mode, which allowed high spatial resolution imaging of the FeRh surface. The images shown in this study were performed over a 1 μm area and at a resolution of 512 × 512 pixels with a scan frequency of 1 Hz. The peakforce setpoint used in this study spanned the range (1–5 nN). Data processing was used to flatten and subtract background planes from the data.

### Data availability

The data that support the findings of this study are available from the corresponding author upon request.

## Additional Information

**How to cite this article:** Barton, C. W. *et al*. Substrate Induced Strain Field in FeRh Epilayers Grown on Single Crystal MgO (001) Substrates. *Sci. Rep.* **7**, 44397; doi: 10.1038/srep44397 (2017).

**Publisher's note:** Springer Nature remains neutral with regard to jurisdictional claims in published maps and institutional affiliations.

## References

[b1] Fallot M. Magnetic properties of iron alloys with ruthenium and osmium. (In French), C. R. Hebd. Seances Acad. Sci. 205, 227–230 (1937).

[b2] Fallot M. Magnetic properties of iron alloys with iridium. (In French), C. R. Hebd. Seances Acad. Sci. 205, 517–518 (1937).

[b3] Fallot M. Magnetic properties of iron alloys with rhodium. (In French), C. R. Hebd. Seances Acad. Sci. 205, 558–560 (1937).

[b4] Hocart R. & Fallot M. The identification of diverse phases by magnetic study and X-rays in alloys of palladium and iron. (In French), C. R. Hebd. Seances Acad. Sci. 204, 1465–1467 (1937).

[b5] Kouvel J. S. Unusual Nature of Abrupt Magnetic Transition in Ferh and Its Pseudobinary Variants. J Appl Phys 37, 1257, doi: 10.1063/1.1708424 (1966).

[b6] Shirane G., Nathans R. & Chen C. W. Magnetic Moments + Unpaired Spin Densities in Fe-Rh Alloys. Phys Rev 134, 1547 (1964).

[b7] Baldasseroni C. . Temperature-driven growth of antiferromagnetic domains in thin-film FeRh. J Phys Condens Matter 27, 256001, doi: 10.1088/0953-8984/27/25/256001 (2015). 26043719

[b8] Vinokurova L. I., Vlasov A. V. & Pardavihorvath M. Pressure Effects on Magnetic Phase-Transitions in FeRh and FeRhIr Alloys. Phys Status Solidi B 78, 353–357, doi: 10.1002/pssb.2220780136 (1976).

[b9] Thiele J. U., Maat S., Robertson J. L. & Fullerton E. E. Magnetic and structural properties of FePt-FeRh exchange spring films for thermally assisted magnetic recording media. IEEE T Magn 40, 2537–2542 (2004).

[b10] Maat S., Thiele J. U. & Fullerton E. E. Temperature and field hysteresis of the antiferromagnetic-to-ferromagnetic phase transition in epitaxial FeRh films. Phys Rev B 72, 214432, doi: ARTN 214432 10.1103/PhysRevB.72.214432 (2005).

[b11] Loving M. . Tailoring the FeRh magnetostructural response with Au diffusion. J Appl Phys 112, 043512, doi: Artn043512 10.1063/1.4747921 (2012).

[b12] Phillips L. C. . Local electrical control of magnetic order and orientation by ferroelastic domain arrangements just above room temperature. Sci Rep-Uk 5, 1–8, doi: ARTN10026 10.1038/srep10026 (2015). PMC4429553 25969926

[b13] Thiele J. U., Buess M. & Back C. H. Spin dynamics of the antiferromagnetic-to-ferromagnetic phase transition in FeRh on a sub-picosecond time scale. Appl Phys Lett 85, 2857–2859, doi: 10.1063/1.1799244 (2004).

[b14] Stamm C. . Antiferromagnetic-ferromagnetic phase transition in FeRh probed by x-ray magnetic circular dichroism. Phys Rev B 77, 184401, doi: ARTN184401 10.1103/PhysRevB.77.184401 (2008).

[b15] Radu I. . Laser-induced generation and quenching of magnetization on FeRh studied with time-resolved x-ray magnetic circular dichroism. Phys Rev B 81, 104415, doi: ARTN104415 10.1103/PhysRevB.81.104415 (2010).

[b16] Naito T., Suzuki I., Itoh M. & Taniyama T. Effect of spin polarized current on magnetic phase transition of ordered FeRh wires. J Appl Phys 109, 07C911, doi: Artn07c911 10.1063/1.3553941 (2011).

[b17] Lee Y. . Large resistivity modulation in mixed-phase metallic systems. Nat Commun 6, 5959, doi: 10.1038/ncomms6959 (2015). 25564764

[b18] Matsuzaki N. . Current induced antiferro-ferromagnetic transition in FeRh nanowires. Jpn J Appl Phys 54, 073002, doi: Artn073002 10.7567/Jjap.54.073002 (2015).

[b19] Moriyama T. . Sequential write-read operations in FeRh antiferromagnetic memory. Appl Phys Lett 107, 122403, doi: Artn122403 10.1063/1.4931567 (2015).

[b20] Marti X. . Room-temperature antiferromagnetic memory resistor. Nat Mater 13, 367–374, doi: 10.1038/nmat3861 (2014). 24464243

[b21] Suzuki I., Itoh M. & Taniyama T. Elastically controlled magnetic phase transition in Ga-FeRh/BaTiO3(001) heterostructure. Appl Phys Lett 104, 022401, doi: Artn022401 10.1063/1.4861455 (2014).

[b22] Toumey C. Plenty of room, plenty of history. Nat Nanotechnol 4, 783–784, doi: 10.1038/nnano.2009.357 (2009). 19966818

[b23] Moore G. E. Cramming more components onto integrated circuits (Reprinted from Electronics, pg 114–117, April 19, 1965). P IEEE 86, 82–85, doi: 10.1109/Jproc.1998.658762 (1998).

[b24] Ayoub J. P., Gatel C., Roucau C. & Casanove M. J. Structure and chemical order in FeRh nanolayers epitaxially grown on MgO(001). J Cryst Growth 314, 336–340, doi: 10.1016/j.jcrysgro.2010.11.127 (2011).

[b25] Han G. C. . Magnetic stability of ultrathin FeRh films. J Appl Phys 113, 17C107, doi: Artn17c107 10.1063/1.4794980 (2013).

[b26] Castiella M. . Structural investigation of magnetic FeRh epitaxial films. Mater Res Express 2, 1–7, doi: Artn086401 10.1088/2053-1591/2/8/086401 (2015).

[b27] Witte R. . Tailoring magnetic frustration in strained epitaxial FeRh films. Phys Rev B 93, 104416, doi: ARTN104416 10.1103/PhysRevB.93.104416 (2016).

[b28] Pressacco F. . Stable room-temperature ferromagnetic phase at the FeRh(100) surface. Sci Rep 6, 22383, doi: 10.1038/srep22383 (2016). 26935274 PMC4776116

[b29] de Vries M. A. . Asymmetric “melting” and “freezing” kinetics of the magnetostructural phase transition in B2-ordered FeRh epilayers. Appl Phys Lett 104, 232407, doi: 10.1063/1.4883369 (2014).

[b30] Fan R. . Ferromagnetism at the interfaces of antiferromagnetic FeRh epilayers. Phys Rev B 82, 184418, doi: ARTN184418 10.1103/PhysRevB.82.184418 (2010).

[b31] Loving M. . Structural evidence for stabilized ferromagnetism in epitaxial FeRh nanoislands. J Phys D Appl Phys 46, 162002, doi: Artn162002 10.1088/0022-3727/46/16/162002 (2013).

[b32] Yang E., Laughlin D. E. & Zhu J. G. Correction of Order Parameter Calculations for FePt Perpendicular Thin Films. IEEE T Magn 48, 7–12, doi: 10.1109/Tmag.2011.2164547 (2012).

[b33] Le Graet C. . Sputter growth and characterization of metamagnetic B2-ordered FeRh epilayers. J Vis Exp, e50603, doi: 10.3791/50603 (2013). PMC3938335 24145690

[b34] Ellis M. O. A. . The Landau-Lifshitz equation in atomistic models. Low Temp Phys 41, 705–712 (2015).

[b35] Barker J. & Chantrell R. W. Higher-order exchange interactions leading to metamagnetism in FeRh. Phys Rev B 92, 094402, doi: ARTN094402 10.1103/PhysRevB.92.094402 (2015).

[b36] Mancini E. . Magnetic phase transition in iron-rhodium thin films probed by ferromagnetic resonance. J Phys D Appl Phys 46, 245302, doi: Artn245302 10.1088/0022-3727/46/24/245302 (2013).

[b37] Gilbert T. L. A phenomenological theory of damping in ferromagnetic materials. IEEE T Magn 40, 3443–3449, doi: 10.1109/Tmag.2004.836740 (2004).

[b38] Ostler T. A., Barton C., Thomson T. & Hrkac G. Modeling the thickness dependence of the magnetic phase transition temperature in thin FeRh films. Physical Review B, 95(6), 064415, https://doi.org/10.1103/PhysRevB.95.064415 (2017).

[b39] Bennett S. P. . Giant Controllable Magnetization Changes Induced by Structural Phase Transitions in a Metamagnetic Artificial Multiferroic. Sci Rep 6, 22708, doi: 10.1038/srep22708 (2016). 26940159 PMC4778125

[b40] Kim J., Ramesh R. & Kioussis N. Revealing the hidden structural phases of FeRh. Phys Rev B 94 (2016).

[b41] Jekal S., Rhim S. H., Hong S. C., Son W. J. & Shick A. B. Surface-termination-dependent magnetism and strong perpendicular magnetocrystalline anisotropy of an FeRh(001) thin film. Phys Rev B 92, doi: 10.1103/PhysRevB.92.064410 (2015).

